# *Leclercia adecarboxylata* Bacteremia in a Patient with Ulcerative Colitis

**DOI:** 10.1155/2014/457687

**Published:** 2014-10-28

**Authors:** Amir Kashani, Morteza Chitsazan, Kendrick Che, Roger C. Garrison

**Affiliations:** ^1^Department of Medicine, Riverside County Regional Medical Center, 26520 Cactus Avenue Moreno Valley, CA 92555, USA; ^2^Division of Gastroenterology and Hepatology, Department of Medicine, Loma Linda University Medical Center, 11234 Anderson Street, Loma Linda, CA 92354, USA

## Abstract

Patients with inflammatory bowel disease (IBD) are a high risk population for bacteremia. Derangement in the mucosal architecture of the gastrointestinal (GI) tract and frequent endoscopic interventions in immunocompromised individuals are considered primary causes. Isolation of opportunistic microorganisms from the bloodstream of IBD patients has been increasingly reported in recent years. *Leclercia adecarboxylata* is a ubiquitous, aerobic, motile, gram-negative bacillus. The human GI tract is known to harbor this rarely pathogenic microorganism. There are only a few case reports of bacteremia with this microorganism; the majority are either polymicrobial or associated with immunocompromised patients. We describe a case of monomicrobial *L. adecarboxylata* bacteremia in a 43-year-old female who presented with bloody diarrhea. Colonoscopy revealed diffuse colonic mucosal inflammation with numerous ulcers, and histopathology revealed crypt abscesses. Following an episode of rectal bleeding, two sets of blood cultures grew *L. adecarboxylata*, which was treated with intravenous ceftriaxone. After a complicated hospital course, she was eventually diagnosed with ulcerative colitis and enteropathic arthritis, treated with intravenous methylprednisolone, mesalamine, and infliximab which resulted in resolution of her symptoms. In our previously immunocompetent patient, derangement of the gut mucosal barrier was the likely cause of bacteremia, yet performing endoscopic intervention may have contributed to bacterial translocation.

## 1. Introduction

*Leclercia adecarboxylata*, formerly known as* Escherichia adecarboxylata*, is a member of the Enterobacteriaceae family, which was first identified by Leclerc in 1962 [1]. Subsequent genomic differentiation from the* Escherichia* genus, in honor of the founder, resulted in the name* Leclercia adecarboxylata* [2]. This is a ubiquitous, aerobic, motile, gram-negative bacillus, which also inhabits the human gastrointestinal (GI) tract [3]. Worldwide, infection with this rarely pathogenic microorganism has been limited to a small number of case reports [4]; of these, only a few reports implicate the GI tract as the focus of infection [5, 6]. We describe a case of* L. adecarboxylata* bacteremia in a patient presenting with bloody diarrhea who was subsequently diagnosed with inflammatory bowel disease (IBD). Such cases might help elucidate risk factors for bacteremia with opportunistic microorganisms in this patient population.

## 2. Case Presentation

A 43-year-old female with a medical history of nonsteroid dependent asthma presented to an outside hospital complaining of one-month frequent bloody diarrhea, body aches, loss of appetite, and 15 kg weight loss. Upon admission she was febrile (39°C); however the rest of the physical examination was reportedly benign. Laboratory findings were remarkable for leukocytosis (18.2 k/mm^3^; reference value: 4.5–11) and hypokalemia (2.6 meq/L; reference value: 3.3–5.3); the hemoglobin level was within normal range (13.9 g/dL; reference value: 12–16 g/dL). Although the initial workup for infectious etiologies was nondiagnostic, she was empirically treated with piperacillin-tazobactam. Two days later, she was transferred to our hospital for a higher level of care. Upon arrival, she was febrile (39.3°C) and tachycardic (125 bpm); moreover, she was found to have polyarthritis, involving the distal joints of her upper extremities. Significant laboratory findings included a low hemoglobin level (8.9 g/dL; reference value: 12–16), hypoalbuminemia (1.2 g/dL; reference value: 3.5–5), elevated C-reactive protein (40.3 mg/L; reference value: <3), and elevated erythrocyte sedimentation rate (104 mm/hr; reference value: <20). Repeated blood cultures in our facility were negative for bacteremia; however antibiotic therapy with piperacillin-tazobactam was continued as the computed tomography scan of the abdomen and pelvis showed evidence of colitis. Studies for infectious colitis were negative. On day three of admission, a peripherally inserted central catheter (PICC) was placed for total parenteral nutrition. One day later, the patient underwent colonoscopy which showed diffuse mucosal inflammation with numerous deep, clean-based ulcers from the rectum to the distal ascending colon. The differential diagnosis included cytomegalovirus (CMV) colitis versus IBD. Empiric ganciclovir was started; however it was discontinued once CMV colitis was ruled out by serology, quantitative polymerase chain reaction, and immunohistochemistry studies. Histopathology revealed subacute colitis, small crypt abscesses, fragments of granulation tissue, and neutrophilic exudate. An extensive rheumatologic workup for her seronegative polyarthritis led to the diagnosis of enteropathic arthritis. On day eight of hospitalization, once the serology for human immunodeficiency virus was confirmed as negative, intravenous methylprednisolone and oral mesalamine were initiated. On the same day, an episode of massive rectal bleeding was reported by the patient. The hemoglobin decreased from 7.8 to 6.7 g/dL and the patient was transferred to the intensive care unit and transfused with packed red blood cells. Two sets of blood cultures obtained at that time showed growth of gram-negative rods in aerobic bottles only. The methylprednisolone was held due to sepsis and the PICC was removed and sent for culture, which was negative. The microorganism was identified as* L. adecarboxylata*, using the fully automated Vitek 2 Compact 60 system (bioMerieux, Inc. Hazelwood, MO). The microorganism showed a pan-sensitive antimicrobial profile (Table 1), so the antibiotic regimen was changed to intravenous ceftriaxone and repeated blood cultures were negative. Therapy with methylprednisolone was resumed, achieving resolution of her bloody diarrhea and partial improvement of her polyarthritis. In light of the aforementioned clinical and paraclinical findings, the patient was diagnosed with ulcerative colitis with severe disease activity (ulcerative colitis disease activity index of 11), responsive to steroids and mesalamine. After a negative workup for latent tuberculosis and completion of the antibiotic course, treatment with infliximab was initiated which resulted in a complete recovery of the polyarthritis and normalization of the inflammatory markers. She was subsequently discharged to an acute rehabilitation facility with a gradual taper of oral prednisone and infliximab infusions every eight weeks.

## 3. Discussion

To date, literature on* L. adecarboxylata* bacteremia has been limited to a few case reports, mostly documented as polymicrobial infections [4, 16]. The majority of these cases have been reported in immunocompromised patients or those with longstanding central venous access. Reported cases of monomicrobial bacteremia with* L. adecarboxylata* in immunocompetent hosts are limited to two cases: one in a patient with a dermal chemical burn [17] and one in an asymptomatic individual with incidental positive blood cultures after blood donation [18]. Our review of the PubMed database revealed only one report implicating the GI tract as the source of* L. adecarboxylata* bacteremia in a patient with peptic ulcer disease (PUD) secondary to chronic use of nonsteroidal anti-inflammatory drugs (NSAIDs) [5]. The authors concluded that PUD created a portal of entry for the bacterium in a patient who had a suppressed immune system due to chronic NSAIDs therapy. A separate report describing the association between the GI tract and* L. adecarboxylata* infection reported the bacteria isolated from a chronically inflamed gallbladder in a patient presenting with abdominal pain [6].

To our knowledge, our patient is the first reported case of monomicrobial* L. adecarboxylata* bacteremia associated with IBD, which occurred in a previously immunocompetent patient. In the setting of IBD, bacteremia has multiple etiologies. Derangement in the mucosal architecture of the gastrointestinal (GI) tract and performing frequent endoscopic interventions in immunocompromised individuals are considered the primary causes [19]. To evaluate the association of IBD and bacteremia with opportunistic microorganisms, we exclusively reviewed such case reports (Table 2). The majority of reported patients were already receiving immunosuppressive agents [7–11]. In some cases, IBD and bacteremia were identified concurrently [12–14]. In this group, bacterial translocation might be as a result of performing invasive interventions on the GI tract with deranged mucosal architecture [12]. However in a few patients, only the mucosal derangement of the gut could be identified as a possible predisposing factor [13–15]. This group of patients had not undergone any invasive interventions prior to the isolation of bacteria from their blood stream. This corroborates the IBD as an independent predisposing factor of bacteremia with opportunistic microorganisms. It has been already shown that derangement in gut mucosal architecture is more evident in patients with active colitis [20]. Therefore, one might hypothesize that these groups are at a higher risk for bacteremia. In our patient, compromised immune status and central venous catheter are unlikely predisposing factors of bacteremia; we believe that the bacteremia occurred as a result of bacterial translocation while performing colonoscopy on an impaired mucosal barrier of the colon in the setting of ulcerative colitis with severe activity.

In conclusion, IBD particularly in severe form is a predisposing factor for bacteremia with opportunistic microorganisms, regardless of the patient's immune status. Invasive interventions on the GI tract must not be overlooked as possible contributory factors to bacterial translocation. Although* L. adecarboxylata* is rarely pathogenic, identifying this microorganism and other such pathogens as potential causes of bacteremia in patients with IBD might help characterize patients at higher risk for bacteremia and guide empiric antimicrobial therapy.

## Figures and Tables

**Table 1:** *Leclercia adecarboxylata* antibiotic susceptibility profile.

Antibiotic	MIC	Susceptibility
Ampicillin	4	S
Cefazolin	≤4	S
Ciprofloxacin	≤0.25	S
Gentamicin	≤1	S
Imipenem	1	S
Levofloxacin	≤0.12	S
Tobramycin	≤1	S
Trimethoprim/sulfamethoxazole	≤20	S

MIC: minimum inhibitory concentration (*μ*g/mL); S: susceptible.

**Table 2:** Bacteremia with opportunistic microorganisms associated with inflammatory bowel disease.

Microorganism	Case	Medication	Intervention^*^	Predisposing factor
*Rothia dentocariosa* [7]	A 42-year-old male with history of UC presents with abdominal pain and bloody diarrhea, subsequently diagnosed with UC flare	Infliximab	PICC	Mucosal derangement; immunosuppression; central venous access

*Desulfovibrio desulfuricans* [8]	69-year-old female with history of OLT due to PSC and UC presents with bloody diarrhea, abdominal pain, and fever, subsequently diagnosed with CMV colitis	Cyclosporine; azathioprine; methylprednisolone	Colonoscopy/biopsy	Mucosal derangement; immunosuppression; endoscopy

*Streptomyces thermovulgaris* [9]	81-year-old woman with history of CD presents with abdominal pain and fever, subsequently diagnosed with CD flare	Prednisone	None	Mucosal derangement; immunosuppression

*Lactobacillus rhamnosus* [10]	17-year-old male with history of UC presents with fever, subsequently diagnosed with bacteremia	Mesalamine; prednisone; infliximab	None	Immunosuppression

*Lactobacillus rhamnosus* [11]	44-year-old woman with history of UC presents with fever and bloody diarrhea (diagnosis not reported)	Prednisone; cyclosporine	Not reported	Mucosal derangement; immunosuppression

*Eggerthella lenta* [12]	21-year-old female presents with abdominal pain, subsequently diagnosed with SBO and CD	None	Complicated ileocolic anastomosis disruption	Mucosal derangement; surgical complication

*Bacteroides fragilis* [13]	44-year-old man with history of bloody diarrhea presents with malaise and fever, subsequently diagnosed with endocarditis and CD	None	None	Mucosal derangement

*Streptococcus gallolyticus* [14]	74-year-old female with history of chronic diarrhea presented with fever and altered sensorium, subsequently diagnosed with meningitis and IBD	None	None	Mucosal derangement

*Fusobacterium nucleatum* [15]	A 56-year-old man with a history of UC presents with fever and jaundice, subsequently diagnosed with PVT	Not reported	None	Mucosal derangement

^*^Any intervention performed prior to blood culture obtained, which may contribute to the bacterial translocation; OLT: orthotopic liver transplantation; PSC: primary sclerosing cholangitis; UC: ulcerative colitis; CMV: cytomegalovirus; SBO: small bowel obstruction; CD: Crohn's disease; PVT: portal vein thrombosis; IBD: inflammatory bowel disease; PICC: peripherally inserted central catheter.
